# Complement Receptors and Their Role in Leukocyte Recruitment and Phagocytosis

**DOI:** 10.3389/fcell.2021.624025

**Published:** 2021-02-11

**Authors:** Sofie Vandendriessche, Seppe Cambier, Paul Proost, Pedro E. Marques

**Affiliations:** Laboratory of Molecular Immunology, Department of Microbiology, Immunology and Transplantation, Rega Institute for Medical Research, Katholieke Universiteit Leuven (KU Leuven), Leuven, Belgium

**Keywords:** complement, complement receptors, leukocyte, inflammation, cell migration, phagocytosis

## Abstract

The complement system is deeply embedded in our physiology and immunity. Complement activation generates a multitude of molecules that converge simultaneously on the opsonization of a target for phagocytosis and activation of the immune system via soluble anaphylatoxins. This response is used to control microorganisms and to remove dead cells, but also plays a major role in stimulating the adaptive immune response and the regeneration of injured tissues. Many of these effects inherently depend on complement receptors expressed on leukocytes and parenchymal cells, which, by recognizing complement-derived molecules, promote leukocyte recruitment, phagocytosis of microorganisms and clearance of immune complexes. Here, the plethora of information on the role of complement receptors will be reviewed, including an analysis of how this functionally and structurally diverse group of molecules acts jointly to exert the full extent of complement regulation of homeostasis.

## Introduction to the Complement System

### Evolution of Complement

The mammalian complement system comprises more than 50 fluid and membrane-associated proteins that control multiple aspects of physiology and immunity (Hajishengallis et al., 2017). The complement system is classically associated to a rapid response to invading microorganisms, which are either opsonized or directly lysed by the proteolytic cascade of complement proteins, as result of the generation of a lytic membrane attack complex (MAC). Complement is a core component of the immune system and is found (in less complex forms) in invertebrates as ancient as corals, jellyfish and sea anemones (phylum Cnidaria), a suggestion that complement may have originated near the appearance of multicellular organisms (Dishaw et al., 2005; Zhang and Cui, 2014). Complement is also found in invertebrates such as snails and clams (phylum Mollusca), insects and arachnids (phylum Arthropoda) and in the amphioxus (phylum Chordata) (Dodds and Matsushita, 2007; Prado-Alvarez et al., 2009; Sekiguchi and Nonaka, 2015). In vertebrates, complement versions resembling the mammalian system are found in jawed fish, such as zebrafish, in which research on complement function and evolution can be performed (Zhang and Cui, 2014). Although discovered first, the classical pathway (see section The Complement Cascade) is the latest in evolutionary terms, since it is closely associated to the function of IgM and IgG antibodies. Instead, the ancestral complement system likely relied on the combined action of prototypic versions of the complement protein C3, Factor B, and a protease (Nakao and Somamoto, 2016). Interestingly, complement in invertebrates and ancestral vertebrates lacks cytolytic activity, which suggests that opsonization was the central role of complement in these animals and that complement-mediated cytolysis appeared later on (Dodds and Matsushita, 2007; Nonaka, 2014).

### The Complement Cascade

**Figure 1:**
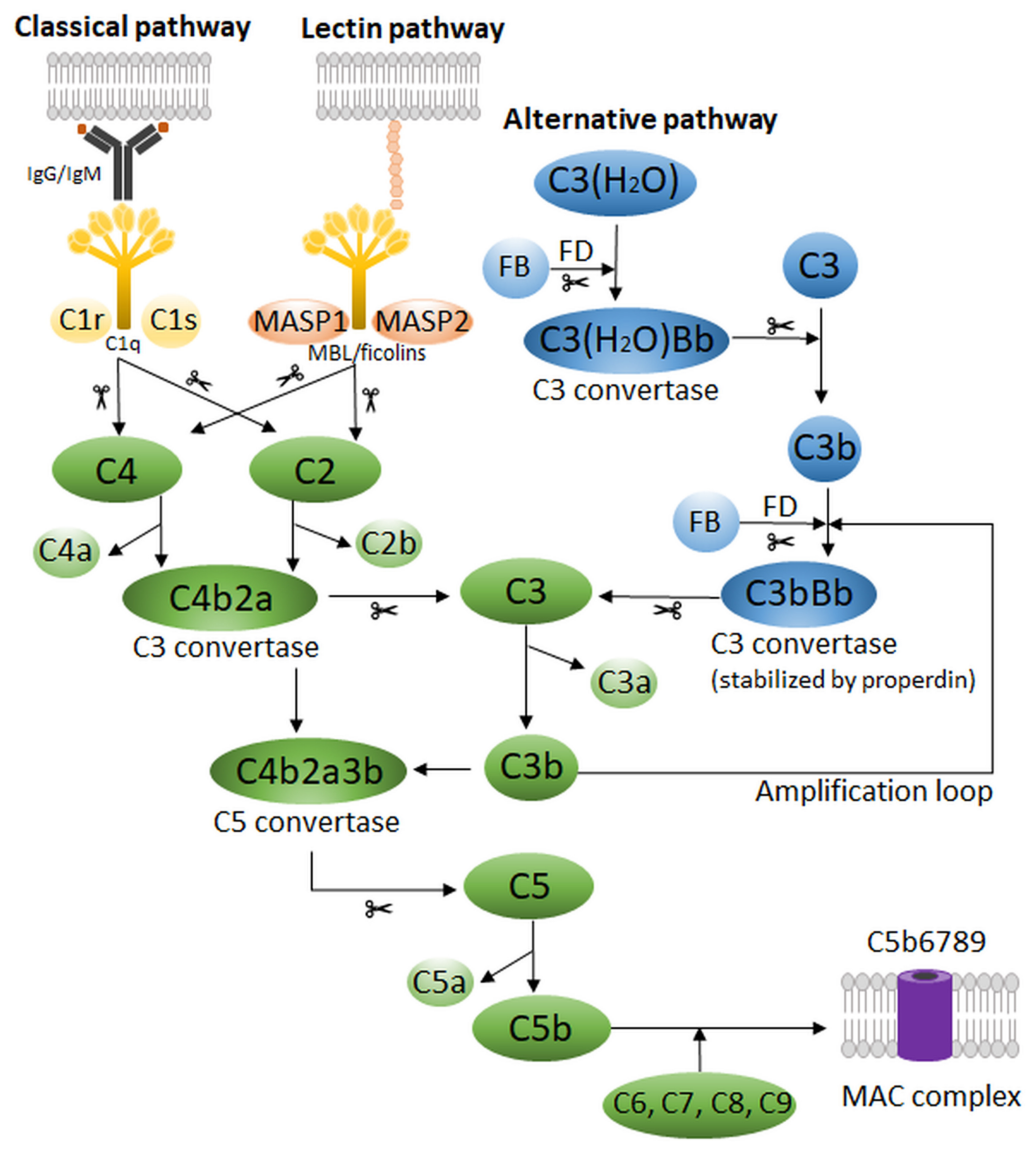
Schematic representation of the human complement system, which possesses three main pathways: the classical, the lectin and the alternative pathway. Scissors indicate proteolytic cleavage. FB, Factor B; FD, Factor D; MAC, membrane attack complex; MASP, MBL-associated serum protease; MBL, mannose-binding lectin.

The complement system in mammals possesses three main pathways: the classical, the lectin and the alternative pathway (Figure 1) (Densen and Ram, 2015). The classical pathway is initiated by complement protein C1q, which can bind to Fc regions of IgM and IgG immune complexes, but also to a variety of antigens directly through its pattern-recognition capabilities (Kouser et al., 2015). Binding of C1q leads to the activation of the C1q-associated proteases C1r and C1s, which will in turn cleave C4 and C2 available in the extracellular fluids. Cleavage of C4 generates C4b, which binds covalently to molecules in the vicinity of the active C1 proteases, associating stably to nearby antigens and to the antibodies. The C2 cleavage fragment C2a associates with C4b to form the C3 convertase, the central step of complement activation. The C3 convertase will cleave thousands of C3 molecules, present abundantly in the blood, into the highly-reactive C3b fragment that associates covalently to neighboring molecules and effectively opsonizes the target (Cooper, 1985). In addition, at this step occurs the release of the other fragment produced during C3 cleavage, the soluble anaphylatoxin C3a, that has pro-inflammatory properties through interaction with its G protein-coupled receptor (GPCR). Subsequently, C3b binding to C3 convertases *in situ* leads to the formation of the C5 convertases, which are responsible for cleavage of C5 into C5b and C5a. As before, C5b associates to the target, although non-covalently, whereas C5a acts as an anaphylatoxin. The remaining components of the complement cascade; C6, C7, C8, and C9 will associate sequentially to the target-bound C5b to form the MAC, which is a pore with cytolytic properties. At its final configuration, the MAC is composed of one copy of C5b, C6, C7, and C8 and up to 18 copies of C9, which constitute the bulk of the MAC pore (Müller-Eberhard, 1986; Merle et al., 2015). The lectin pathway differs from the classical mainly because it is driven by mannose-binding lectin (MBL) and ficolins (Figure 1). Analogously to C1q, they bind to antigens to initiate the complement cascade, but in this case, the antigens are carbohydrates such as mannose, glucose and N-acetyl-glucosamine (Holers, 2014; Merle et al., 2015). Binding of MBL to sugars on microbial membranes leads to activation of MBL-associated serum proteases (MASPs) 1 and 2, which structurally and functionally similar to C1r and C1s, will initiate the cleavage of C4 and C2 to form the C3 convertase and unleash the complement cascade.

The complement cascade can also be initiated spontaneously by C3, the so-called alternative pathway (Figure 1). In the fluid phase, C3 can undergo spontaneous low-rate hydrolysis, or “tick-over,” to yield C3(H_2_O) (Holers, 2014; Hajishengallis et al., 2017). This form can bind Factor B, which when cleaved by the protease Factor D, produces a fluid-phase C3 convertase [C3(H_2_O)Bb]. This enzyme produces C3b in solution, which may deposit on nearby surfaces and complex with more Factor B, forming the C3 convertase of the alternative pathway (C3bBb). It is important to mention that this cascade is short-lived and insufficient to promote full complement activation. For that, the alternative pathway requires properdin, a phagocyte-derived protein that stabilizes C3bBb and allows it to cleave C3 for long enough to enter the amplification phase of complement activation. Interestingly, the alternative pathway does not require antibodies for initiation, however, the presence of antibodies, polysaccharides, lipopolysaccharides (LPS), gas bubbles, heme, and properdin are all known to facilitate its activation. Moreover, the alternative pathway contributes to the full activation of complement via all pathways (e.g. classical and lectin) by providing an “amplification loop,” in which deposited C3b continuously forms new C3 convertases by binding to Factor B. This dramatically increases the cascade activity and is associated to its effects *in vivo*, including complement-mediated injury (Holers, 2014; Hajishengallis et al., 2017).

### Complement Regulation

Considering the high concentration of complement components in the serum and the ability of the cascade to self-amplify, a number of inhibitory mechanisms and molecules were developed throughout evolution in order to regulate the effects of complement on host cells (Ricklin et al., 2010; Holers, 2014; Densen and Ram, 2015; Merle et al., 2015). The initiation of the cascade is regulated by the C1 inhibitor (C1-INH/SERPIN 1), which inactivates the C1 proteases and dissociates them from C1q. Also, C1-INH inhibits several other proteases, including MASPs, thus regulating the lectin pathway. It is worth noting that the C3 convertases are unstable and undergo decay spontaneously, stopping the cascade unless stimuli are present. There are multiple soluble molecules that accelerate the deactivation of convertases, namely: Factor I, a protease that cleaves C3b into the enzymatically inactive form iC3b; Factor H, which accelerates the dissociation of Bb from C3 convertases and facilitates Factor I cleavage of C3b; C4 binding protein (C4BP), which stimulates the dissociation of C2a from C4b (classical C3 convertase) and also acts as co-factor for Factor I-mediated cleavage of C4b into iC4b. Additionally, the soluble proteins clusterin (SP-40) and vitronectin (S protein) bind to nascent C5b-C9 complexes and inhibit MAC assembly. Importantly, the activity of anaphylatoxins C3a and C5a is regulated via cleavage by carboxypeptidase-N, a plasma zinc metalloprotease, which cleaves a C-terminal arginine residue that severely reduces the anaphylatoxins' inflammatory effects. There are also a number of membrane-associated complement inhibitors. They serve to restrain the deleterious effects of excessive complement activation, but also offers a means to separate healthy host cells from other complement targets, such as microorganisms, dead cells and crystals, which most often do not express complement inhibitors. These include: Membrane cofactor protein (MCP, CD46), which binds to C3b and C4b fragments and stimulate Factor I-mediated cleavage; Decay-accelerating factor (DAF, CD55), which dissociates C3 convertases by binding to C3b and C4b; CR1 (CD35), that shares both dissociation and cofactor activities of MCP and DAF; and CD59, which binds C8 and C9 to prevent MAC assembly.

### Complement-Associated Diseases and Therapies

Taking into account its ancient origin and broad reach, it is not surprising that complement regulates central aspects of physiology and immunity. Deficiencies in the complement system predispose individuals to severe recurrent infections and higher incidence of autoimmune disorders as systemic lupus erythematosus (SLE) (Ricklin et al., 2010; Holers, 2014). Conversely, deficiencies in the complement regulatory proteins DAF, MCP, and factor H lead to paroxysmal nocturnal hemoglobinuria and atypical hemolytic uremic syndrome, conditions in which complement attacks and destroys red blood cells and endothelial cells, respectively. Moreover, complement activation has been implicated in the progression of rheumatoid arthritis and Alzheimer's disease, driven by, respectively, recognition of immune complexes in the joints and amyloid deposits in the brain. Interestingly, it was demonstrated recently that complement gene variants also control the incidence of disorders between males and females (Kamitaki et al., 2020). It was shown that higher levels of C4 and C3 in men are correlated to their higher risk of developing schizophrenia, whereas the lower levels of these proteins in women are correlated to their substantial predisposition to develop SLE. Likewise, a number of treatments targeting complement-mediated diseases have been developed and are currently undergoing clinical trials (Ricklin et al., 2019). Complement inhibitors are being evaluated in a variety of kidney diseases (Zipfel et al., 2019), brain injury and neurodegenerative disorders (Brennan et al., 2016), rheumatic diseases (SLE and rheumatoid arthritis) (Trouw et al., 2017), and transplantation (Biglarnia et al., 2018; Thorgersen et al., 2019). Notably, Eculizumab and Ravulizumab (anti-C5 monoclonal antibodies), CCX168 (C5aR receptor antagonist), purified/recombinant C1-INH, and AMY-101 (C3 cleavage inhibitor) have undergone successful phase II/III trials for several diseases. For an extensive review on complement inhibitors in clinical trials see (Mastellos et al., 2019).

Although complement is a self-sustaining cascade with effector functions, the biological effects of complement depend largely on complement receptors. Complement receptors are functionally and structurally diverse, which goes along with their varied roles in leukocyte activation, recruitment, adhesion and phagocytosis. This article aims to review the plethora of information on the role of complement receptors in these functions, with focus on receptor expression, structure and ligand recognition, and connections to effector functions in leukocytes and human disease.

## Role of Complement Receptors in Leukocyte Recruitment

Acute inflammation is a reaction of the host to tissue damage or infection by microorganisms. The inflammatory response is usually beneficial, as it will try to resolve this pathological condition by neutralizing damaging agents so that homeostasis can be restored. Due to recognition of pathogen-associated molecular patterns (PAMPs) or damage-associated molecular patterns (DAMPs), pro-inflammatory chemical mediators will be produced and released by tissue-resident cells at the inflammatory site. These mediators including cytokines, chemokines, histamine, prostaglandins and leukotrienes will not only provoke the classical inflammatory symptoms of erythema, heat, pain, and edema but also leukocyte chemotaxis and extravasation into the surrounding inflamed tissue. Once the leukocytes have arrived at the inflammatory site, they can neutralize the inflammatory trigger [by phagocytosis, production of reactive oxygen species (ROS), degranulation, etc.], hence stressing the importance of the leukocyte recruitment process (Medzhitov, 2008). In this section of the review, we will specifically focus on the role of complement receptors in this recruitment process. First, we will focus on the complement anaphylatoxins and their receptors, followed by the role of integrins in leukocyte adhesion and extravasation into the inflamed tissue.

### Complement Anaphylatoxins

**Figure 2:**
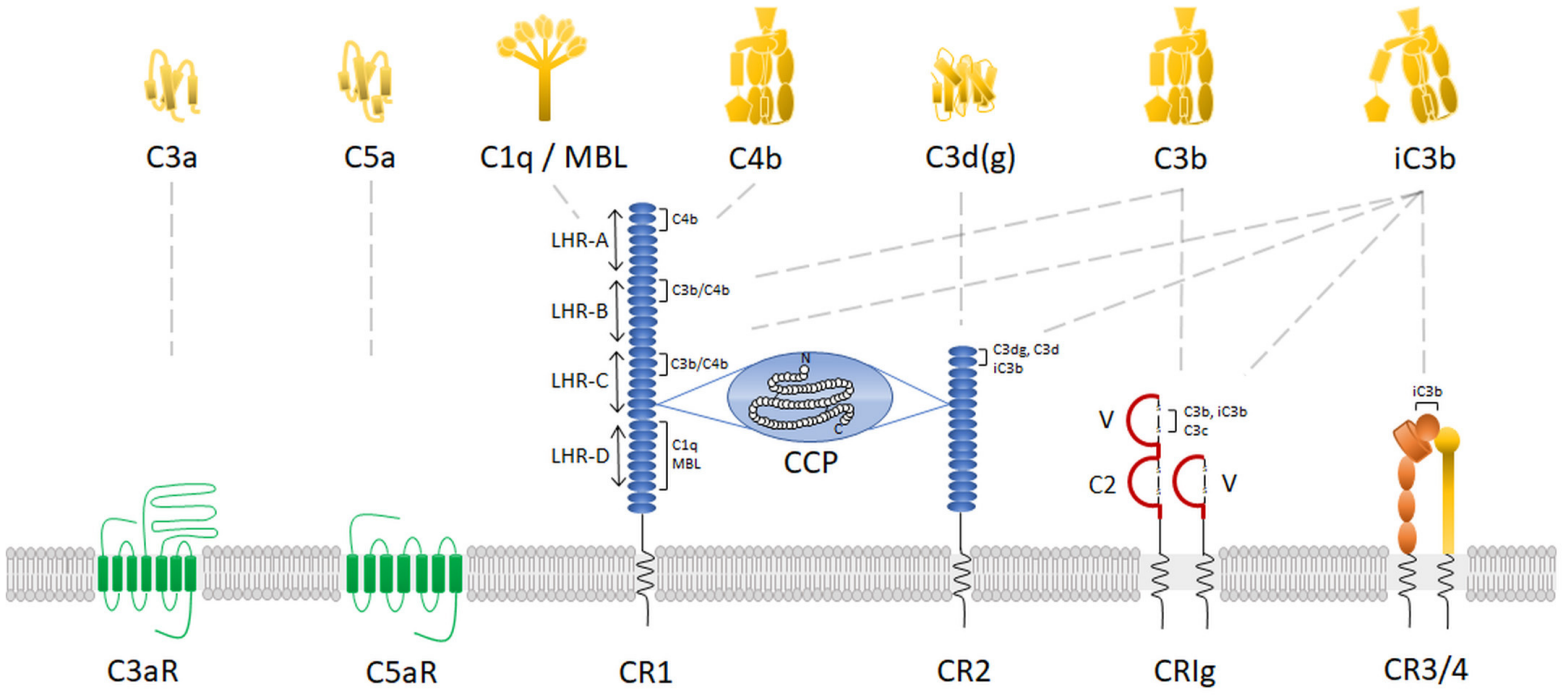
Complement receptors and their main ligands. Schematic representation of human complement receptors on the plasma membrane with their corresponding complement protein ligands. For CR1, CR2, CRIg, and CR3/4, the binding areas for each specific ligand are indicated. In addition, for CR1, the receptor domains are identified at the left side of the receptor. CCP, complement control protein repeats; LHR, long homologous repeats; MBL, mannose-binding lectin.

Complement fragments C3a and C5a are small anaphylatoxins, mediating pro-inflammatory effects by binding to their respective G protein-coupled complement receptors C3aR and C5aR. Both human C3a (1–77 amino acids) and C5a (1–74 amino acids) are structurally composed of a core of four α-helices stabilized by three disulfide bonds and connected by loop segments (Figure 2) (Huber et al., 1980; Zuiderweg et al., 1989; Zhang et al., 1997). C-terminally in C3a, there is a flexible, cationic, irregular structure (Hugli, 1975; Huber et al., 1980) from which the five final C-terminal amino acids, LGLAR, form the active site of C3a. More specifically, the hydrophobic side chains of leucine-73 and leucine-75 and the guanidinium group of arginine-77 are key in the active site (Caporale et al., 1980). In C5a, the α-helical bundle core is connected with a small loop to the five final C-terminal amino acids, that adopt an α-helical conformation (Zhang et al., 1997). Interestingly, and in contrast to C3a, C5a contains a complex carbohydrate chain N-linked to asparagine-64 (Fernandez and Hugli, 1978). The activity of C3a and C5a is tightly controlled; Carboxypeptidase-N cleaves the carboxy-terminal arginine from both C3a and C5a in the bloodstream, reducing their biological activity 10-100 fold [reviewed in (Matthews et al., 2004)]. It is noteworthy that complement fragment C4a is also considered an anaphylatoxin. Although structurally similar to C3a and C5a (Moon et al., 1981), it lacks an identified complement receptor and its functional capacities are poorly characterized (Barnum, 2015). Recently, it was shown that C4a can act as a ligand for protease-activated receptor (PAR)1 and PAR4, affecting endothelial permeability. However, no role for C4a in direct leukocyte chemotaxis has been described (Wang et al., 2017).

#### C3aR

##### Receptor characterization

C3aR is a cell surface complement receptor part of the GPCR family, first cloned by Ames et al. (1996) and Crass et al. (1996). Its gene *C3AR*, only comprising one exon located on chromosome 12p13 (Paral et al., 1998), shares 37% nucleotide identity throughout the coding regions with C5aR. C3aR is not a classical GPCR as it has an unusually large second extracellular loop (~172 amino acids) between transmembrane domains 4 and 5 (Figure 2) (Ames et al., 1996). Deletion mutagenesis studies showed that multiple aspartate residues in the loop, adjacent to the transmembrane domains, are essential for C3a binding and the following downstream mobilization of intracellular Ca^2+^ (Ames et al., 1996; Chao et al., 1999). Indeed, they provide a secondary interaction site through electrostatic interaction with cationic residues in the C-terminal helical region of C3a (Hugli, 1975; Chao et al., 1999). The primary ligand effector binding site in C3aR, identified to be mainly formed by charged residues in a cluster of transmembrane helices (Sun et al., 1999), is engaged by the active site of C3a being the C-terminal sequence LGLAR (Caporale et al., 1980; Chao et al., 1999). Interestingly, the natural C3a catabolite C3a(desArg) has no binding affinity and cannot activate C3aR (Wilken et al., 1999).

##### Expression and function in leukocyte recruitment

The transcript of C3aR is widely expressed in peripheral tissues such as the lung, spleen, ovary, placenta, small intestine, heart, peripheral blood leukocytes and in the central nervous system (Ames et al., 1996). C3aR expression could be identified on neutrophils, basophils, eosinophils, monocytes and mast cells through flow cytometry, northern blotting, calcium release and binding assays (Table 1) (Hartmann et al., 1997; Martin et al., 1997; Zwirner et al., 1999). It was shown however that C3aR expression on monocytes and neutrophils is about 6 to 20 times lower than C5aR expression, respectively (Zwirner et al., 1999). Receptor expression has not been demonstrated on unchallenged B-lymphocytes and T-lymphocytes (Martin et al., 1997; Zwirner et al., 1999) but expression has been shown on activated T-lymphocytes (Werfel et al., 2000) and on tonsil-derived B-lymphocytes (Fischer and Hugli, 1997). Besides leukocytes, expression of C3aR on bronchial epithelial and smooth muscle cells of the lung has been shown (Drouin et al., 2001). In the central nervous system, C3aR is expressed in astrocytes and microglia during inflammation (Gasque et al., 1998). Moreover, constitutive expression has also been shown on neurons, which suggests a role in physiological conditions (Davoust et al., 1999). In the rat, it was already shown that C3aR plays a role in the development of the cerebellum (Bénard et al., 2008).

**Table 1:** Expression, function and main ligands of complement receptors.

**Receptor**	**Alternative name**	**Main ligands**	**Main leukocyte expression**	**Main functions**
C3aR	/	C3a	Neutrophils Basophils Eosinophils Monocytes Mast cells Activated T-lymphocytes Tonsil-derived B-lymphocytes	Chemotaxis of mast cells, eosinophils and monocytes/macrophages (Daffern et al., 1995; Hartmann et al., 1997; Zwirner et al., 1998) Induction of ROS production in neutrophils (Elsner et al., 1994) No (or inhibition of) chemotaxis of neutrophils (Daffern et al., 1995; Wu et al., 2013) Retention of hematopoietic stem cells in the bone marrow (Reca et al., 2003; Ratajczak et al., 2004) Leukocyte recruitment to the brain (Wu et al., 2016; Crider et al., 2018) Regeneration of skeletal muscle and hepatic tissue (Strey et al., 2003; Markiewski et al., 2004; Zhang et al., 2017)
C5aR	C5aR1	C5a	Neutrophils Monocytes/macrophages Dendritic cells T-lymphocytes B-lymphocytes Mast cells	Chemotaxis of neutrophils, monocytes, dendritic cells, T- and B-lymphocytes (Morgan et al., 1993; Sozzani et al., 1995; Nataf et al., 1999; Ottonello et al., 1999) Degranulation of neutrophils (Morgan et al., 1993) Induction of ROS production in neutrophils (Sacks et al., 1978) Cytokine production in monocytes (Morgan et al., 1993) Mast cell histamine release (Johnson et al., 1975; Füreder et al., 1995) Liver regeneration (Mastellos et al., 2001; Daveau et al., 2004; Marshall et al., 2014)
C5L2	C5aR2	C5a	Immature dendritic cells Granulocytes (Myeloid immune cells) T cell subsets	Immune suppressing and immune activating functions due to regulation of C5aR activation and signaling (Li et al., 2019)
CR1	CD35 C3b/C4b receptor	C1q C3b C4b iC3b MBL	Erythrocytes Monocytes/macrophages Granulocytes B-lymphocytes CD4+ T-lymphocytes FDCs Glomerular podocytes	Immune regulatory role (Iida and Nussenzweig, 1981; Masaki et al., 1992) Immune-complex clearance (Cornacoff et al., 1983) Phagocytosis (Fällman et al., 1993)
CRIg	VSIG4/B7 family- related protein Z39Ig	C3b C3c iC3b	Tissue-resident macrophages MDDCs	Complement-mediated phagocytosis (Wiesmann et al., 2006) Immune regulatory role (Vogt et al., 2006; Munawara et al., 2019)
CR2	CD21	iC3b C3dg C3d gp350/220 (EBV) CD23 Interferon-alpha	B-lymphocytes FDCs T-lymphocytes Epithelial cells	Lowering B-lymphocyte activation threshold (Carter and Fearon, 1992) Retention of C3-opsonized antigens (Reynes et al., 1985) Promotion of B-lymphocyte class switching and IgE production (Aubry et al., 1992) EBV cell entry (Tanner et al., 1987)
CR3	Mac-1 CD11b/CD18 Integrin α_M_β_2_	ICAM-1 ICAM-2 Fibrinogen iC3b Collagen Factor X NIF	Neutrophils Monocytes/macrophages Dendritic cells NK cells Activated lymphocytes	Leukocyte extravasation (Dustin and Springer, 1988; Meerschaert and Furie, 1995; Ding et al., 1999; Phillipson et al., 2006) Phagocytosis (Beller et al., 1982)
CR4	p150,95 CD11c/CD18 Integrin α_X_β_2_	ICAM-1 ICAM-2 Fibrinogen iC3b Collagen Factor X NIF	Neutrophils Monocytes/macrophages Dendritic cells NK cells Activated lymphocytes	Adhesion to fibrinogen (Sándor et al., 2016) Phagocytosis (Keizer et al., 1987)

*EBV, Epstein-Barr virus; FDCs, follicular dendritic cells; ICAM, intercellular adhesion molecule; Mac-1, macrophage-1 antigen; MBL, mannose-binding lectin; MDDCs, monocyte-derived dendritic cells; NIF, neutrophil inhibitory factor; NK, natural killer; ROS, reactive oxygen species; VSIG4, V-set and Ig domain 4*.

C3aR activation is associated with G protein-coupled downstream signaling and an increase in intracellular Ca^2+^, which is blocked by pertussis toxin (Norgauer et al., 1993; Elsner et al., 1994). Activation of the receptor induces dissociation of the Gα (primarily Gα_i_) subunit and the G_βγ_ subunit. Gα_i_ inhibits adenylyl cyclase, resulting in a reduced intracellular cyclic adenosine monophosphate (cAMP) concentration. G_βγ_ activates phospholipase Cβ (PLCβ), leading to increased intracellular Ca^2+^ and protein kinase C (PKC) activation. In addition, it activates phosphoinositide 3-kinase γ (PI3Kγ) resulting in activation of extracellular signal–regulated kinases (ERKs) and phosphokinase B (Akt) (Futosi et al., 2013). This signaling cascade, has been shown to induce chemotaxis of mast cells (Hartmann et al., 1997) and eosinophils (Daffern et al., 1995). Induction of macrophage chemotaxis by C3a was first shown in the mouse macrophage cell line J774 (Zwirner et al., 1998). Activation of C3aR does not induce direct neutrophil chemotaxis (Fernandez et al., 1978; Elsner et al., 1994; Daffern et al., 1995). However, C3a was able to induce ROS production in neutrophils (Elsner et al., 1994) and C3aR activation also induced formation of neutrophil extracellular traps (NETosis), leading to hypercoagulation and tumor-promoting effects *in vivo* (Guglietta et al., 2016). Wu et al. reported a role for C3aR as inhibitor of neutrophil mobilization and protection from intestinal ischemia-reperfusion injury. Indeed, C3aR^−/−^ mice had an augmented number of tissue-infiltrating and circulating neutrophils, which were associated to worsening of intestinal damage, whereas stimulation of C3aR in WT mice reduced neutrophil mobilization and consequent intestinal injury (Wu et al., 2013). An interesting study showed a role for C3a in the retention of hematopoietic stem cells in the bone marrow. C3a-C3aR interaction can counteract mobilization of hematopoietic stem cells by increasing their response to stromal-derived factor 1 (SDF-1/CXCL12), of which the expression decreases in the bone marrow during mobilization (Reca et al., 2003; Ratajczak et al., 2004). In the central nervous system, it was shown using C3^−/−^ and C3aR^−/−^ mice that C3a plays a role in cerebral endothelial activation (by upregulation of adhesion molecules) and leukocyte recruitment to the LPS-inflamed brain (Wu et al., 2016). Also, a specific link between C3a and depression was found. In mice, it was shown that C3a induces monocyte infiltration into the prefrontal cortex after exposure to chronic stress, which is specifically associated with depressive-like behavior (Crider et al., 2018). Interestingly, C3a-C3aR interaction promotes monocyte recruitment into inflamed skeletal muscle. There, it plays an essential role during regeneration of skeletal muscle, as this regeneration can be impaired by C3a inactivation or C3aR deletion (Zhang et al., 2017). C3a is also involved in the regeneration of hepatic tissue after injury. After partial hepatectomy in C3^−/−^ mice, normal liver regeneration was impaired, which was associated with clinical deterioration and higher mortality compared to WT mice (Strey et al., 2003). Moreover, liver regeneration was impaired after a toxic challenge in C3^−/−^ mice. This could however be reversed by administration of C3a and activation of C3aR (Markiewski et al., 2004).

#### C5aR

##### Receptor characterization

C5aR (C5aR1 or CD88) is a cell surface GPCR first discovered in 1991 (Figure 2) (Gerard and Gerard, 1991). The gene *C5AR1* comprises two exons and is located on chromosome 19, band position q13.3 (Gerard et al., 1993). C5aR is bound by its ligand complement fragment C5a according to a two-site binding model (Siciliano et al., 1994). The extracellular N-terminal portion of C5aR [with required aspartic acids (DeMartino et al., 1994) and sulfations of N-terminal tyrosines (Farzan et al., 2001)] is essential in the formation of the docking site for the core of C5a via electrostatic interactions (Mery and Boulay, 1994; Siciliano et al., 1994). Interaction of C5a with the N-terminal part of the receptor is required for high affinity binding and full activation of the receptor (DeMartino et al., 1994). However, just as in the C3a-C3aR interaction, the primary ligand binding site is located between the transmembrane helices at the base of the C5aR extracellular loops. This binding site interacts with the C-terminal part of C5a (Siciliano et al., 1994). In line with this, C-terminal peptide fragments of C5a are sufficient to activate C5aR, even if the receptor is N-terminally truncated. It is believed that the interaction between C5a and the N-terminal part of C5aR is essential to induce a conformational change in C5a, which allows its C-terminal part to interact with and to activate the receptor (DeMartino et al., 1994). Interestingly, the highest residue homology with C3aR is found in the transmembrane domains and in the second intracellular loop (Ames et al., 1996). Later on, it was shown via receptor mutagenesis that arginine-206 located in the 5th transmembrane helix of C5aR is essential for high-affinity binding to C5a (Raffetseder et al., 1996).

##### Expression and function in leukocyte recruitment

The C5aR transcript is co-expressed with C3aR in several peripheral tissues such as the lung, spleen, placenta and the central nervous system. C5aR is expressed to a higher extent than C3aR in peripheral blood leukocytes and the heart (Table 1) (Ames et al., 1996). More specifically, its expression was described on human polymorphonuclear leukocytes in Chenoweth and Hugli (1978) and on murine macrophages in Chenoweth et al. (1982). Nevertheless, induction of directed chemotaxis of polymorphonuclear leukocytes by C5a had already been shown *in vitro* and in simulated *in vivo* conditions before the receptor was known (Shin et al., 1968; Fernandez et al., 1978). Later on, with the use of polyclonal antibodies directed against the extracellular N-terminal part of C5aR, inhibition of C5a-mediated neutrophil chemotaxis and -degranulation, and inhibition of cytokine production by monocytes was demonstrated (Morgan et al., 1993). Furthermore, C5a can induce production of ROS in granulocytes, which resulted in endothelial cell cytotoxicity *in vitro* (Sacks et al., 1978). Moreover, C5a-C5aR interaction plays a specific role in neutrophil and monocyte migration to the synovium of rheumatoid and psoriatic arthritis patients (Hornum et al., 2017). In a mouse model of autoantibody-induced inflammatory arthritis, activation of C5aR on neutrophils in the joint vasculature perpetuates their own recruitment. Indeed, C5a production due to complement opsonization on immune complexes in the joints, results in release of the chemotactic lipid leukotriene B4 (LTB4) from arrested neutrophils which promote further neutrophil migration to the interstitium (Sadik et al., 2018). Functional expression of C5aR has also been shown on dendritic cells (Sozzani et al., 1995) and skin mast cells. Expression of C5aR was specifically associated with histamine release after C5a stimulation (Johnson et al., 1975; Füreder et al., 1995). Conversely, expression of C5aR on human T-lymphocytes was relatively low, but T-lymphocytes are responsive to a C5a gradient when receptor expression is increased by phytohemagglutinin stimulation (Nataf et al., 1999). Ottonello et al. also showed low expression of C5aR on naive and memory B-lymphocytes, which was sufficient to promote a response to recombinant C5a *in vitro* (Ottonello et al., 1999). However, on murine (un)stimulated T- or B-lymphocytes no expression of C5aR was observed, in contrast to granulocytes and macrophages (Soruri et al., 2003).

Besides leukocytes, C5aR is expressed in several cell types of the lung (lung vascular smooth muscle, endothelium, bronchial and alveolar epithelium) and in liver parenchymal cells (Haviland et al., 1995; Drouin et al., 2001). Together with C3a, C5a-C5aR interaction is required in liver regeneration. Indeed, C5^−/−^ mice display abnormal liver regeneration after partial hepatectomy or toxic injury (Mastellos et al., 2001; Strey et al., 2003). Moreover, mice deficient in both C3 and C5 have an even more severe defect in regeneration, which can be partially reversed by reconstitution with C3a or C5a. Administration of both anaphylatoxins together led to a better recovery, suggesting that C3a and C5a act cooperatively in the early priming stages of hepatocyte regeneration. Furthermore, in a rat model of partial hepatectomy, C5aR was upregulated on hepatocytes promoting their regrowth after injury (Daveau et al., 2004). Blockade of C5aR reduces the intrahepatic release of interleukin-6 (IL-6) and tumor necrosis factor alpha (TNF-α). However, in contrast to their inflammatory roles in most tissues, these cytokines are essential for hepatocytes to reenter the cell cycle and initiate liver regeneration via activation of the transcription factors nuclear factor kappa-light-chain-enhancer of activated B cells (NF-κB) and signal transducer and activator of transcription 3 (STAT-3) (Strey et al., 2003). Interestingly, the site-targeted murine complement inhibitor CR2-CD59 (which specifically inhibits the assembly of the MAC) is able to promote hepatocyte proliferation, and so, liver regeneration after hepatectomy. CR2-CD59 can increase the intrahepatic IL-6 and TNF-α levels, resulting in STAT-3 and Akt activation required for liver regeneration (Marshall et al., 2014).

#### C5L2

Human C5a receptor-like 2 (C5L2 or C5aR2) is a seven-transmembrane spanning receptor related to the C5a and C3a receptor, first discovered in 2000 in immature dendritic cells and granulocytes (Ohno et al., 2000). Expression was also described in the bone marrow, spleen, lung, in most myeloid immune cells and T cell subsets, and it is present both intracellularly and on the cell surface (Li et al., 2019). C5L2 is able to bind C5a and hence functions as a second C5a receptor (Okinaga et al., 2003). However, it lacks the ability to induce downstream signaling due to an amino acid (R→L) replacement in the DRY motif. This highly conserved motif, located at the end of the third transmembrane segment, is necessary for GPCR interaction with G proteins. As a consequence, C5L2 is unable to induce downstream signaling and calcium increase, and it may function as a decoy receptor (Okinaga et al., 2003). Several studies however reported C5L2 as an important regulator of C5aR activation and downstream signaling. Bamberg et al. indicated that C5L2 might function as a negative modulator of ERK1/2 signal transduction through modulation of β-arrestin after C5aR activation by C5a. Inhibition of C5L2 resulted in increased C5a-mediated chemotaxis, but no alterations in C5a-induced Ca^2+^-responses (Bamberg et al., 2010). It was also shown that C5L2 can physically interact with C5aR, forming heterodimers (Croker et al., 2013). Thus, C5a stimulation may trigger C5aR–C5L2 heterodimerization and β-arrestin recruitment, facilitating C5aR internalization and downregulating C5aR-mediated ERK signaling (Croker et al., 2014; Li et al., 2019). In contrast, it has been shown that C5L2 also exerts stimulatory functions: On the endothelium, it promotes C5a translocation into the blood vessel lumen, mediating neutrophil arrest through C5aR activation in a murine arthritis model (Miyabe et al., 2019). More elaborated information about the current knowledge of this controversial receptor and its role in pathophysiology was recently reviewed by Li et al. (2019).

### β_2_-Integrin Family Complement Receptors CR3 and CR4

#### Receptor Characterization

In contrast to the chemotactic receptors C3aR and C5aR, β2-integrins are heterodimeric cell surface adhesion receptors that play a crucial role in cell adhesion, migration and communication. They are involved in cell-cell and cell-extracellular matrix interactions and have the unique capacity to mediate bidirectional transmission of mechanical and biochemical signals across the membrane. Structurally, integrins are characterized by a non-covalent association of two type I membrane glycoproteins, the α and β subunit, consisting of a small cytoplasmic tail (<75 amino acids, except for the β4 subunit), a transmembrane region and a large extracellular domain containing ligand-binding sites (>100 kDa for α subunits and >75 kDa for β subunits) (Hynes, 1992). The subunits form together an extracellular domain composed of a N-terminal globular ligand-binding head and a C-terminal tailpiece, formed by two long “legs” or “stalks,” connecting with the transmembrane and short cytoplasmic domains of each subunit (Nermut et al., 1988) (Figure 2). The N-terminal region of the α-domain contains seven segments of about 60 amino acids, which fold into a seven-bladed β-propeller domain that forms the globular head region of the receptor. About half of the integrins, more specifically the leukocyte β_2_-integrins and the integrin collagen receptors, have an approximately 200 amino acid insertion in between the second and third beta sheet that is known as the inserted (I) domain. This domain contains a metal ion-dependent adhesion site (MIDAS) for binding of divalent cations required for ligand binding (Diamond et al., 1993; Michishita et al., 1993; Lee et al., 1995; Tuckwell et al., 1995). The leukocyte β_2_-integrin family includes four members that share a common β_2_-subunit (CD18) linked to one of four α-chains: αLβ_2_-integrin [also referred to as lymphocyte function-associated antigen 1 (LFA-1) or CD11a/CD18], αMβ_2_-integrin [macrophage-1 antigen (Mac-1), CR3 or CD11b/CD18], αXβ_2_-integrin [p150,95; CR4 or CD11c/CD18] and αDβ_2_-integrin [CD11d/CD18] (Springer et al., 1979; Kürzinger et al., 1981; Sanchez-Madrid et al., 1983; Van der Vieren et al., 1995). Within the β2-integrin family, there are two complement receptors: CR3 and CR4 (Table 1).

#### Expression and Function in Leukocyte Recruitment

**Figure 3:**
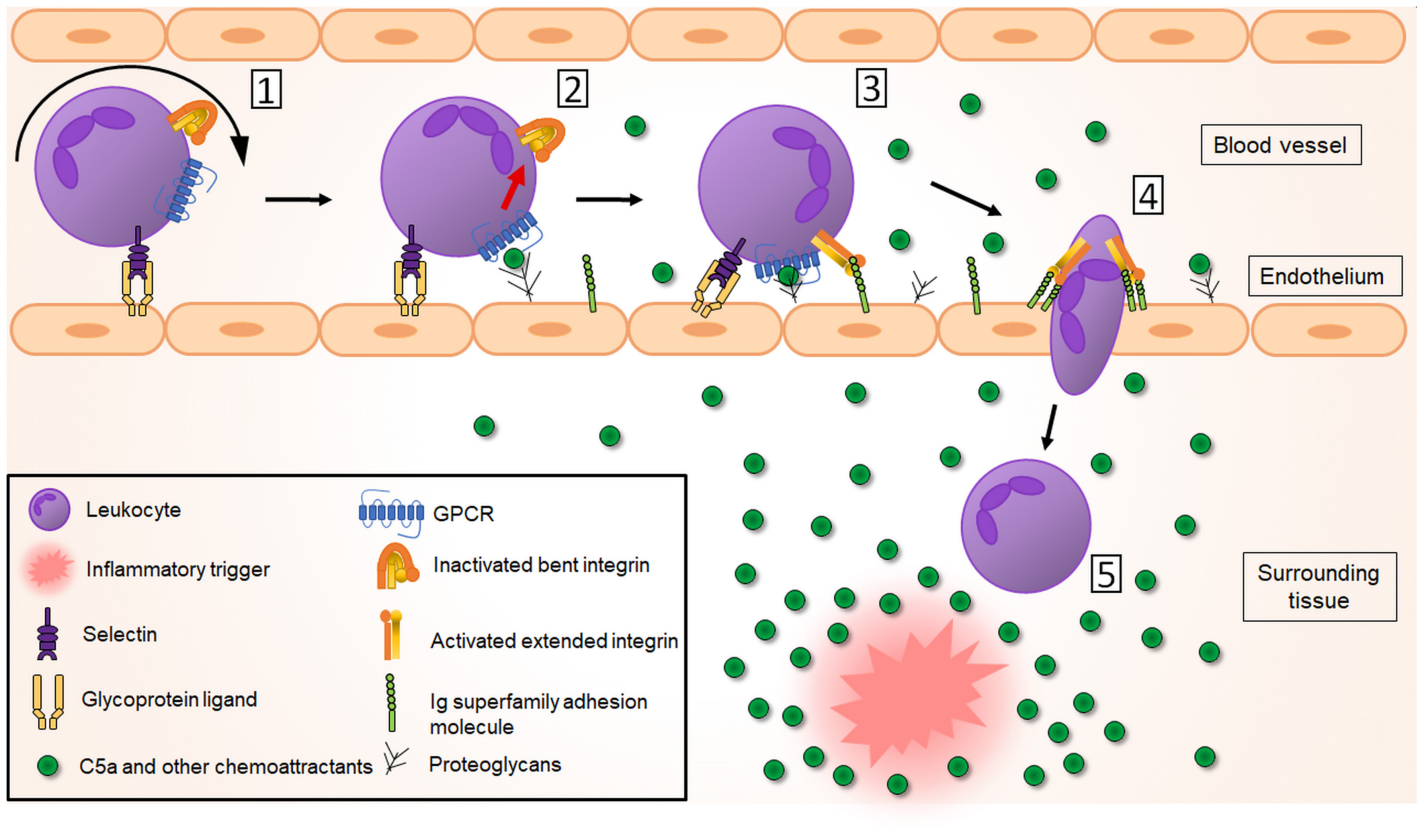
Complement receptors play a crucial role in leukocyte recruitment to the inflammatory site. [1] In response to an inflammatory trigger (infection/tissue damage), leukocytes first roll over the activated endothelium through weak selectin-glycoprotein interactions (for clarity only leukocyte selectins and endothelial glycoproteins are shown here). [2] This allows interaction of leukocyte GPCRs (including C5aR but also other chemoattractant receptors) with chemoattractant molecules, produced by tissue-resident cells in response to and forming a chemotactic gradient toward the inflammatory trigger. This results in inside-out activation of integrins, changing their global conformation from a bent non-activated conformation to an extended, activated conformation with higher affinity for the integrin ligands ICAM-1, ICAM-2 and VCAM-1, which belong to the immunoglobulin (Ig) superfamily adhesion molecules [3]. This leads to a tight adhesion and arrest of the leukocytes to the endothelium. After integrin-mediated crawling to an optimal emigration spot directed by the chemotactic gradient, a transmigratory cup is formed and leukocytes transmigrate [4] through the endothelial barrier and the basement membrane into the surrounding tissue, where their migration will be guided further to the inflammatory site directed by the chemoattractant gradient [5].

Leukocyte recruitment to tissues is an essential step in the inflammatory response that requires the binding and extravasation of leukocytes in the vasculature. In the muscle and intestines, it starts with rolling of the leukocyte over the activated endothelium, followed by leukocyte activation and firm adhesion, diapedesis through the endothelial layer and further migration into the tissue matrix (Figure 3) (Muller, 2013). Integrins play a crucial role in this complex and tightly controlled process. In steady state, passively moving blood leukocytes express bent, non-activated “resting” integrins. In this conformation, the ligand-binding head domain of the integrin is folded over the tailpiece, moving the ligand binding site close to the C-terminal, membrane-proximal end of the tailpiece (“legs” of the integrin). This is an unfavorable orientation for ligand binding and consequently bent integrins have only low affinity for their endothelial ligands, including the immunoglobulin superfamily members intercellular adhesion molecule 1 and 2 (ICAM-1 and -2) and vascular cell adhesion molecule 1 (VCAM-1) (Xiong et al., 2001, 2002; Takagi et al., 2002; Chen et al., 2010a). In response to microbial infection or tissue damage, release of pro-inflammatory mediators increases the blood flow and the expression of endothelial adhesion molecules. This allows leukocytes to increase rolling along the luminal side of the activated endothelium, mediated by weak and transient selectin-glycoprotein interactions (McEver, 2002). These interactions can already change the integrin conformation to a more extended one which supports slower leukocyte rolling, as shown for the integrin LFA-1 in neutrophils (Kuwano et al., 2010; Stadtmann et al., 2013). Indeed, binding of P-selectin glycoprotein ligand-1 (PSGL-1) to endothelial selectins leads to activation of Src kinases and Ras-related protein 1 (Rap-1) [see below], supporting slow rolling (Yago et al., 2018). In monocytes, interaction between the β1-integrin very late antigen-4 (VLA-4; integrin α4β1) and VCAM-1 supports transition to slow rolling followed by firm adhesion (Huo et al., 2000).

Due to slow rolling, leukocytes can be activated through interaction with chemokines, C5a, platelet-activating factor (PAF) or LTB4, which are present in the endothelial vicinity or found bound to proteoglycans on the endothelial surface (Middleton et al., 1997; Lefort and Ley, 2012; Miyabe et al., 2017). Leukocyte activation then induces integrin activation via inside-out signaling (also called priming), in which intracellular signals initiated by the GPCR activation are transduced to the cytoplasmic domain and further to the extracellular part of the integrin. A global conformational change in the integrin structure (via a switchblade-like opening) will result in less than a second in an extended, activated, higher affinity state of the integrin. The inside-out signaling process was recently reviewed by Bednarczyk et al. (2020). In short, GPCR-mediated activation of the small GTPase Rap-1 causes it to colocalize with the effector Rap1-GTP-interaction adapter molecule (RIAM). RIAM recruits the cytoskeletal protein talin, which is required for the unbending of the two integrin subunits. The N-terminal globular head of talin contains a four-point-one, ezrin, radixin, moesin (FERM) domain that interacts with the conserved NPXY motifs in the β cytoplasmic integrin domain, thereby disrupting the α and β tail salt bridge to generate the high affinity conformation which enables ligand binding (Calderwood et al., 2002; Campbell and Ginsberg, 2004). Also, the adapter protein Kindlin-3 is able to bind to NPXY motifs and contributes to the full integrin activation (Moser et al., 2008). Eventually, the extended, higher affinity state of the integrin results in a firm adhesion and arrest of the leukocytes to the endothelium (Takagi et al., 2002; Xiao et al., 2004; Nishida et al., 2006). More details on structural rearrangements of integrins is provided by Luo et al. (2007). Due to binding of ligands to the integrins and consequent outside-in signaling, the conformation of the integrin can again slightly change resulting in even higher affinity, further strengthening and stabilizing leukocyte adhesion (Takagi et al., 2002) which has been shown for the interaction between LFA-1 and ICAM-1 (CD54) (Chen et al., 2010b). Moreover, Src kinases are involved in sustaining the adhesion of neutrophils through outside-in signaling. Indeed, lack of this outside-in signaling leads to rapid detachment of adherent neutrophils from the endothelium (Giagulli et al., 2006).

Once leukocytes arrest, intraluminal crawling is performed toward optimal emigration sites nearby endothelial cell borders (Massena et al., 2010). In neutrophils, it was shown using intravital microscopy that this crawling mechanism is dependent on interaction between CR3 and endothelial ICAM-1 (Diamond et al., 1991; Phillipson et al., 2006) and ICAM-2 (Halai et al., 2014). Of note, binding of LFA-1 to ICAM-1 and VLA-4 to VCAM-1 has also been shown to contribute to neutrophil adhesion (Staunton et al., 1990; Reinhardt et al., 1997; Ding et al., 1999; Phillipson et al., 2006). In monocytes, however, the primary integrin involved in monocyte adhesion and arrest is VLA-4, due to its interaction with VCAM-1 (Hyduk et al., 2007), with a secondary role for LFA-1 and CR3 (Meerschaert and Furie, 1995). In addition, in monocytes both LFA-1 (Auffray et al., 2007) and CR3 are involved in crawling via interactions with ICAM-1 and ICAM-2 (Schenkel et al., 2004; Sumagin et al., 2010). In lymphocytes, LFA-1 binding to ICAM-1 on endothelial cells is required for lymphocyte adhesion (Dustin and Springer, 1988), but on stimulated endothelium, also interaction between VLA-4 and VCAM-1 was observed (Elices et al., 1990; Vennegoor et al., 1992). The actual crossing of leukocytes through the endothelial layer (diapedesis) into the tissue happens via a transcellular or a paracellular route (Carman and Springer, 2004). Integrins are also involved in this process. To initiate diapedesis, a “transmigratory cup” on the endothelial surface is formed by redistribution of leukocyte integrins LFA-1, CR3 and VLA-4. They colocalize with clusters of ICAM-1 and VCAM-1 on endothelial microvilli-like projections at the junctional interface between the adherent leukocyte and the endothelium (Carman and Springer, 2004; Shaw et al., 2004). In neutrophils, also ICAM-2 seems to play a role as genetic deletion or blockade of ICAM-2 partially inhibits the neutrophil transmigration process (Huang et al., 2006). ICAM-2 mediates neutrophil transmigration in a stimulus-dependent manner, alongside other adhesion molecules (Woodfin et al., 2009). For more details on transendothelial migration see (Filippi, 2019) and (Gerhardt and Ley, 2015).

Once beyond the endothelial layer, leukocytes still have to cross a discontinuous layer of pericytes, which are long cells surrounding the endothelium and embedded within the basement membrane. The interaction of CR3 and LFA-1 with ICAM-1 on the pericytes is necessary for abluminal crawling of neutrophils, guiding them to gaps (exit points) into the interstitium (Proebstl et al., 2012). This seems to happen specifically at regions poor in extracellular matrix proteins, such as sites low on laminins and collagen IV (Wang et al., 2006). A slightly different process is performed by monocytes. Monocytes were shown to be more deformable, so they do not need to remodel the basement membrane to enlarge these low expression regions, as neutrophils do (Voisin et al., 2009). Using intravital imaging, it has been shown that individual neutrophils, once inside the locally inflamed tissue, can show highly coordinated chemotaxis forming neutrophil clusters (“swarms”). This migration process in extravascular spaces was shown in several mouse models and tissues. “Neutrophil swarming” occurs during infection with bacteria, fungi or parasites, as well as during sterile inflammation (Kienle and Lämmermann, 2016; Lämmermann, 2016). Neutrophil swarming can be of a transient or persistent nature (Chtanova et al., 2008) and its phenotype is influenced by the size of the initial tissue damage, the presence of pathogens, the number of recruited neutrophils and induction of secondary cell death (Kienle and Lämmermann, 2016). Long-distance migration in the neutrophil “swarms” is integrin independent (Lämmermann et al., 2008), but integrin adhesive forces are required to maintain the dense neutrophil clusters. These allow neutrophils to accumulate in the wound center, excluding collagen fibers and making a collagen-free zone. Based on knock out of neutrophil integrins, it was identified that both LFA-1 and CR3 are important to maintain the cell adhesion in the neutrophil “swarm” (Lämmermann et al., 2013).

The role of CR4 (CD11c/CD18) in cellular adhesion is less well characterized. CR4 is closely related to CR3: the entire CR4 α-chain (CD11c) shares 63% sequence homology to the CR3 α-chain (CD11b) (Corbi et al., 1988). Both receptors recognize similar ligands such as iC3b, fibrinogen and ICAMs and so it was believed they also mediate similar functions. However, expression of CD11c is dominating in monocyte-derived macrophages (MDMs) and monocyte-derived dendritic cells (MDDCs) where the ratio with CD11b expression is close to 1:1. In circulating monocytes, in contrast, CD11c expression is about 7 times lower than CD11b. Functionally, it was shown that CR4 is the main receptor for strong adhesion to the extracellular matrix component fibrinogen. Although CD11b can also bind to fibrinogen, it was shown that blockade of CD11c strongly reduces adhesion strength whereas blockade of CD11b enhances the attachment of MDDCs and MDMs to fibrinogen. Thus, CD11b can have a competitive, negative role in adhesion of MDDCs and MDMs to fibrinogen (Sándor et al., 2016).

Defects in the synthesis of the common β-chain (CD18) of integrins LFA-1, CR3 or CR4 lead to the rare autosomal-recessive disease leukocyte adhesion deficiency (LAD)-I, characterized by absence or reduced expression of these integrins on leukocytes. As a result, patients have deficiencies in leukocyte adhesion and abnormalities in several adherence-dependent functions like chemotaxis and aggregation. LAD patients are susceptible to recurrent bacterial infections and impaired wound healing, amongst other symptoms, and often die during childhood (Anderson and Springer, 1987; Hogg et al., 1999). A LAD-I-like phenotype is also seen in LAD-III, another autosomal-recessive disease characterized by mutations in Kindlin-3, which is required in the inside-out signaling and activation of β2-integrins (Stepensky et al., 2015). However, LAD-II which yields similar immunodeficiency, is not directly related to integrin functions. Instead, in LAD-II, mutations in a specific GDP-fucose transporter result in impaired synthesis of selectin glycoprotein ligands, impairing the leukocyte rolling and eventually resulting in impaired leukocyte extravasation (Sturla et al., 2001).

## Role of Complement Receptors in Phagocytosis

### Introduction to Phagocytosis

**Figure 4:**
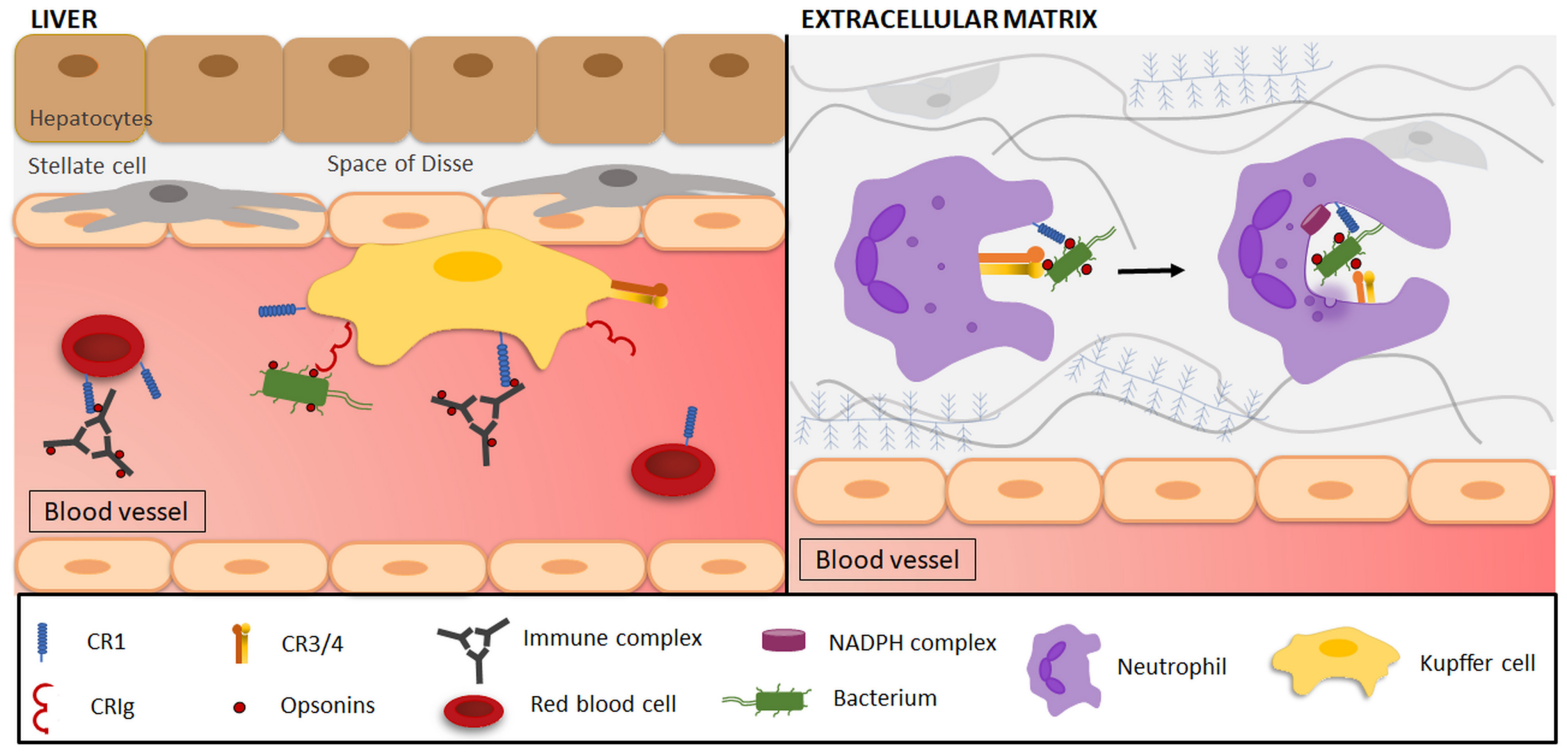
Complement-mediated phagocytosis in the clearance of microorganisms and immune complexes. (Left side) Resident liver macrophages (Kupffer cells/KCs) engulf pathogens, immune complexes and various other particles through interactions with complement receptors. Opsonized (red dots) immune complexes (ICs) are transported to the liver via CR1 on the plasma membrane of circulating red blood cells (RBCs). In the liver, ICs interact with CR1 and scavenger receptors (not shown) on KCs, leading to IC phagocytosis, where after RBCs return to the circulation. Opsonized and free pathogens are recognized by CRIg on KCs and are subsequently engulfed. (Right side) Phagocytes (e.g., neutrophils) are attracted to a site of infection and reach the extracellular matrix by transmigrating across the endothelium. Once there, CR1 on neutrophils will be involved in the attachment of C3-opsonized particles to the cell, after which interaction with CR3 mediates phagocytosis. As the plasma membrane surrounds the target, forming a phagosome, the activation of the nicotinamide adenine dinucleotide phosphate (NADPH) complex and degranulation of neutrophilic granules initiate target degradation.

Phagocytosis is a cellular process characterized by the recognition and ingestion of particles larger than 0.5 μm into a membrane-encased vesicle, the phagosome (Nordenfelt and Tapper, 2011; Flannagan et al., 2012). This process contributes to tissue homeostasis and remodeling, and participates in the host defense as it eliminates microorganisms and foreign substances (Figure 4). Classical phagocytosis is initiated by the interaction of a particle with specific receptors on the surface of professional phagocytes, more specifically, macrophages and neutrophils. Those receptors include non-opsonic phagocytic receptors, such as Dectin-1 and Mincle that directly recognize conserved PAMPs. Moreover, other receptors recognize host-derived opsonins attached to pathogens, such as complement (e.g., CR3) and Fc gamma receptors (FcγRs), which are extensively studied phagocytic receptors for opsonized particles. FcγRs bind the conserved Fc domain of immunoglobulins (Ig), which causes receptor clustering and phosphorylation of immunoreceptor tyrosine-based activation motifs (ITAMs) in the cytoplasmatic tail of the receptor, leading to the recruitment of Syk kinases. This activates various downstream signaling pathways (e.g., ERK, phospholipase D, PKC) mediating cell effector functions such as actin-dependent pseudopod extensions of the plasma membrane around the particle to draw it into the cell (May and Machesky, 2001). In contrast, studies using electron microscopy showed that little to no membrane protrusions were formed during complement-mediated internalization of an opsonized particle, as the particle appeared to “sink” into the cell (Griffin et al., 1975; Kaplan, 1977). The more recent idea, validated by live-cell imaging of the entire phagocytic process, is that actin-based membrane protrusions (pseudopods) are formed that surround iC3b-opsonized beads, a mechanism that could have been previously missed during electron microscopy processing (Hall et al., 2006; Patel and Harrison, 2008; Rotty et al., 2017; Jaumouille et al., 2019). This active, phagocytic cup-mediated internalization of the complement-coated particle has become a consensus in the field, replacing the older particle “sinking” model.

Activation of complement receptors leads to signaling pathways and actin cytoskeleton reorganization in such a way that the membrane completely surrounds the target particle, thereby forming the phagosome. The phagocytic cup formation is primed by integrin inside-out signaling induced by Toll-like receptor (TLR) or GPCR stimulation (Freeman and Grinstein, 2014). Talin recruitment induces the high-affinity, extended integrin conformation that enables ligand binding (Calderwood et al., 2002; Campbell and Ginsberg, 2004). Talin also mediates the mechanical coupling of integrins to actin filaments, as forces arising during phagocytosis expose vinculin binding sites on talin (del Rio et al., 2009). This will promote focal adhesion kinase (FAK)-mediated tyrosine phosphorylation of paxillin together with vinculin binding to talin and actin, which serves as a “molecular clutch” that drives phagocytosis (Jaumouille et al., 2019). In turn, integrin “outside-in” signaling will promote actin reorganization to form plasma membrane protrusions. This formation is driven by the Arp2/3 complex, nucleating actin filaments branches from the sides of pre-existing filaments. The activity of the Arp2/3 complex is indispensable during complement-mediated phagocytosis, since Arp2/3 inhibition with CK-666 inhibits phagocytosis (May et al., 2000; Rotty et al., 2017; Jaumouille et al., 2019). This activity depends on the activation of the GTPase Rho in complement phagocytosis, while GTPases Cdc42 and Rac are required in Fc-mediated phagocytosis (Caron and Hall, 1998). Rho recruits and stimulates the formin mDia, which drives actin protrusions to the particle surface and connects the actin cytoskeleton to microtubules (Palazzo et al., 2001). Active Rho also enables actomyosin contractions by activating the Rho kinase (ROCK), responsible for the increased myosin light chain phosphorylation (Olazabal et al., 2002). Syk kinase activity is required for vinculin recruitment and strengthening of force transmission for optimal particle uptake. Once the target particle is internalized, it combines with early endosomes coming from the endoplasmic reticulum and Golgi apparatus to form the early phagosome. The early phagosome is characterized by the presence of Rab5 and EEA1 proteins at the phagosomal membrane, and does not increase in size, as recycling endosomes are removed from the phagosome and trafficked back to the plasma membrane. Upon phagosome maturation, the fusion with late endosomes modifies the now called late phagosome, which is characterized by the presence of Rab7 and the incorporation of additional copies of the vacuolar-type H+-ATPase (V-ATPase). Finally, lysosomes fuse with the late phagosome to become phagolysosomes which have an acidic pH, and contain hydrolytic enzymes and nicotinamide adenine dinucleotide phosphate (NADPH) oxidase to produce ROS that jointly degrade the ingested particle (Allen and Aderem, 1996).

The importance of complement opsonization in phagocytosis has been demonstrated by a number of studies. C3-deficient mice are immunocompromised and susceptible to lethal bacterial infections. Moreover, clearance of bacteria in the bloodstream by Kupffer cells (KCs) (Helmy et al., 2006) and in the lungs by alveolar macrophages (Neupane et al., 2020) is impaired in C3-deficient mice. Other examples include the reduced phagocytic clearance of microorganisms in the presence of serum that has been depleted of complement by heat-inactivation (Scribner and Fahrney, 1976) or treatment with cobra venom factor (Shin et al., 1969). Cobra venom factor has been extensively used *in vitro* and *in vivo*: It binds to complement Factor B of the alternative pathway to form a venom factor-Bb complex that functions as a C3/C5 convertase (Cooper, 1973; von Zabern et al., 1980). This results in the cleavage of C3 and C5, consequently consuming complement components and ultimately leading to the depletion of serum complement activity. The key role of complement opsonization in phagocytosis was also demonstrated by the interference of complement activation by pathogenic virulence factors. In the case of gram-positive *S. pneumoniae*, the viral capsule inhibits IgG binding and decreases bacterial opsonization with iC3b, preventing phagocytosis by FcR and complement receptors (Hyams et al., 2010). Also, the *E. coli* capsule may block complement opsonization by masking surface components (such as LPS) capable of activating the complement pathway (Horwitz and Silverstein, 1980). Impairing complement opsonization of bacteria is not only a defense mechanism that impairs phagocytosis, but also NETosis. In general, complement opsonization has been proven to promote NETosis, as shown by the enhanced NET release by neutrophils in the presence of the serum-opsonized bacteria *A. actinomycetemcomitans* (Palmer et al., 2016). Yet, this effect was not observed with serum-treated *S. Aureus*, suggesting again that complement-inactivating properties of bacteria might impair effector functions such as phagocytosis and NETosis.

Inherited deficiencies of early classical complement proteins are closely associated with the development of SLE. This systemic autoimmune disease is characterized by antinuclear antibodies, disturbed complement activation and the occurrence of large immunocomplexes (ICs). Homozygous deficiency of C1q is associated with a 93% risk of developing SLE. In addition, 75% of patients with homozygous C4 deficiency develop lupus-like illness (Lewis and Botto, 2006). SLE was observed in 10–30% of C2-deficient patients, indicating a higher risk of SLE when deficiencies occur in complement proteins of earlier stages of the cascade. How complement deficiency exactly contributes to the development of SLE is not known. One possible mechanism is impairment of complement-mediated immune complex clearance (Figure 4), which leads to the deposition of large non-soluble complexes in tissues. These ICs drive proinflammatory cytokine release and local tissue injury (Davies et al., 1992, 1994). This mechanism is supported by the lower expression of CR1 found on erythrocytes of SLE patients, which is the main receptor involved in IC clearance (Ross et al., 1985). Also, the impaired clearance of apoptotic cells due to complement deficiency might serve as a source of self-antigens to initiate autoimmunity. This is evidenced by the impaired phagocytosis of dead/necrotic cells by macrophages in the absence of C1q (Böttcher et al., 2006; Gullstrand et al., 2009).

### Complement Receptor 1 (CR1)

#### Receptor Characterization

Complement receptor 1 (CR1, CD35, C3b/C4b receptor) is a type 1 membrane bound glycoprotein that specifically interacts with MBL and complement proteins C1q, C3b, C4b, and with a lower affinity with iC3b (Figure 2 and Table 1) (Nelson, 1953; Gigli and Nelson, 1968; Ghiran et al., 2000). Four polymorphic variants of the receptor exist, the most common one having a molecular mass of approximately 220 kDa and an extracellular domain of 30 tandemly repeating complement control proteins (CCPs), also known as short consensus repeats or sushi domains. The CCP repeats are 59–72 amino acids long, each having four conserved cysteines that form a pattern of disulfide bridges connecting Cys1-Cys3 and Cys2-Cys4 (Reid et al., 1986; Ahearn and Fearon, 1989; Hannan et al., 2005). The CCPs are further grouped into 4 longer homologous repeats (LHR) containing each 7-9 CCPs. There are several binding sites for C3b and C4b: CCPs 1-3 (LHR-A) contains a C4b binding site, whereas CCPs 8–10 (LHR-B) and CCPs 15–17 (LHR-C) contain both C4b and C3b binding sites, although C3b will bind with a higher affinity (Klickstein et al., 1988; Krych et al., 1991). LHR-D is responsible for the binding of two other CR1 ligands, C1q and MBL. CR1 is expressed on erythrocytes, monocytes/macrophages, polymorphonuclear leukocytes, B-lymphocytes, subpopulations of T-lymphocytes, follicular dendritic cells (FDCs) and glomerular podocytes (Fearon, 1980; Reynes et al., 1985; Appay et al., 1990; Rødgaard et al., 1991). The soluble form of CR1 (sCR1) is released in the plasma by cell surface proteolytic cleavage of CR1 on leukocytes, and acts together with membrane-bound CR1 as an inhibitor of the classical and alternative complement pathway (Danielsson et al., 1994).

#### CR1-Mediated Functions

Protection of host cells against complement activation is mediated by a group of cell surface anchored regulatory proteins, to which CR1 belongs to. CR1 prevents unintended complement-mediated injury by decay-accelerating activity of both C3 and C5 convertases and by serving as a co-factor of the serine protease factor I. This promotes factor I-mediated degradation of C3b and C4b, and the cleavage of iC3b to C3c and C3dg (Iida and Nussenzweig, 1981; Masaki et al., 1992). Besides its regulatory function, CR1 also plays a critical role in the clearance of complement-coated ICs (Figure 4). Formation of ICs by binding of multiple antibodies to antigens leads to C3b deposition, causing the opsonized IC to bind to CR1 on erythrocytes. The ICs are subsequently transported to the liver and spleen on the plasma membrane of circulating erythrocytes, where they interact with Fc-receptors and CR1 on macrophages, leading to IC phagocytosis (Cornacoff et al., 1983; Yoshida et al., 1986). After IC delivery, erythrocytes return to the circulation partially lacking CR1 due to proteolytic cleavage during phagocytosis by macrophages, however, CR1 loss on erythrocytes might also be a consequence of erythrocyte maturation (Pascual et al., 1994; Imrie and Jones, 1997; Miot et al., 2002). Ninety-five percent of the CR1 receptors in the peripheral blood circulation are located on erythrocytes, even though the absolute amount of CR1 on erythrocytes is remarkably lower than on neutrophils (950 vs. 57,000 receptors per cell, respectively). However, the high number of circulating erythrocytes makes the clearance of C3b-opsonized ICs by erythrocytes 500–1,000 times more likely than by leukocytes (Siegel et al., 1981).

CR1 activity also modulates humoral immunity since it facilitates the retention of antigens to FDCs in the germinal center within secondary lymphoid organs. CR1 on FDCs will capture complement-opsonized immune complexes that carry antigens, which stimulate follicular B-lymphocytes via the B-cell receptor (BCR) (Fang et al., 1998). Deficiency in C3 or depletion of circulating C3 with cobra venom factor inhibited memory B-lymphocyte formation and showed the indispensable role of C3 opsonization of ICs for appropriate B-lymphocyte function (Klaus and Humphrey, 1977). In addition, a CR1 knock-out mouse model showed a reduced amount of activated B-lymphocytes in the germinal center and a decreased antibody response (Donius et al., 2013).

In mice, CR1 and CR2 are derived from alternative splicing of the *Cr2* gene, while in humans they are a product of two distinct but closely linked genes on chromosome 1: *CR1* and *CR2* [extensively reviewed by (Jacobson and Weis, 2008)]. In addition, CR1 and CR2 expression in mice is limited to B-lymphocytes and FDCs, complicating the use of a mouse model to investigate CR1 functions on myeloid cells. In mice, CR1 still possesses binding sites for C3b and C4b and serves as a cofactor for C3b cleavage by murine factor I, however, its prominent role in phagocytosis and immune adherence is absent (Kinoshita et al., 1985; Molina et al., 1994). Instead, CR1 on FDCs enhances the retention of antigens on their surface to generate an appropriate antibody response by activated B-lymphocytes of the germinal center, as discussed above (Donius et al., 2013). Interestingly, rodents carry the Cr1-related protein Y (*Crry*) gene, from which the human *CR1* gene has evolved, encoding a membrane-bound complement regulatory protein that is expressed in almost every cell type. Thus, it explains the high degree of protein sequence similarity with human CR1, which is translated to a similar effector function, since it also accelerates the decay of C3/C5 convertases and acts as a cofactor for factor I-mediated cleavage of C3b and C4b (Kim et al., 1995). However, the involvement in phagocytosis and adhesion has not been demonstrated yet for *Crry*, indicating that the mouse homolog for CR1's immune adherence and phagocytosis roles is still unidentified.

The limited research into the phagocytic role of human CR1 has mostly been conducted in the 1980s. The adhesive and phagocytic function of CR1 were mainly assessed through rosette or cluster formation of immunoglobulin and complement-coated sheep erythrocytes to polymorphonuclear leukocytes, monocytes and macrophages, and their subsequent ingestion. The phagocytosis of C3b/C4b-opsonized particles occurs in synergy with CR1 and Fc-receptors on both human and murine neutrophils and macrophages (Mantovani, 1975; Ehlenberger and Nussenzweig, 1977). CR1 is primarily involved in the attachment of C3b-opsonized particles to the cell as shown by the impaired attachment of C3b-coated erythrocytes after anti-CR1 treatment (Newman et al., 1985), whereafter interaction with the Fc-receptors or CR3 mediates phagocytosis (Figure 4) (Fällman et al., 1993). C3 opsonization by itself is not able to trigger CR1-mediated phagocytosis, therefore the presence of IgG complexes is essential to stimulate particle ingestion through its Fc fragments (Newman et al., 1984). CR1 phagocytosis is only mediated after receptor transition from a resting state, in which it binds ligand-coated particles, to an activated state. Even though the biochemical events that account for the shift in activity are unknown, an important role for CR1 phosphorylation has been suggested as PKC stimulation with phorbol myristate acetate (PMA) and PAF enabled phagocytic function by CR1 (Changelian and Fearon, 1986; Bussolino et al., 1989). Likewise, the signaling pathway used by activated CR1 to mediate its effector functions has not yet been characterized.

### Complement Receptor of the Immunoglobulin Family (CRIg)

#### Receptor Characterization

In 2000, a novel human *Z39Ig* gene on chromosome X was reported, encoding a new member of the immunoglobulin superfamily (Langnaese et al., 2000). A few years later, the complement receptor of the immunoglobulin family (CRIg), also referred to as V-set and Ig domain (VSIG4)/B7 family-related protein or Z39Ig, was identified as a receptor expressed on tissue resident and sinusoidal macrophages, especially hepatic KCs, and more recently also on human monocyte derived dendritic cells (Table 1) (Helmy et al., 2006; Munawara et al., 2019). CRIg is a type 1 transmembrane receptor with two alternatively spliced variants in humans: long huCRIg(L) and short huCRIg(S) (Figure 2). The latter consists only of an extracellular variable (V-type) Ig domain, while huCRIg(L) also contains a constant (C2-type) Ig domain. Mice express only one form of muCRIg with a single Ig V-type domain and therefore resembles the shorter human splice variant.

#### CRIg-Mediated Functions

CRIg binds to the beta chain of C3b, to iC3b and C3c, and this receptor is required for the binding and phagocytosis of opsonized pathogens from the circulation, thereby limiting systemic bacteremia or parasitemia (Wiesmann et al., 2006). This has been shown by the reduced capture and elimination of *S. aureus* and *L. monocytogenes* by the liver KCs in CRIg knock-out mice compared to wild-type mice (Helmy et al., 2006). Also, complement opsonization of parasites has been shown to be indispensable for capture and clearance via CRIg by KCs (Liu et al., 2016). However, other investigators observed that complement depletion did not affect the capture of gram-positive bacteria, suggesting that CRIg may bind microorganisms directly in a complement-independent manner (Zeng et al., 2016). This led to the discovery that CRIg functions as a pattern recognition receptor that recognizes gram-positive bacteria via lipoteichoic acid binding *in vitro*. Nevertheless, whether this occurs under high shear forces *in vivo* was questionable (Zeng et al., 2016). Moreover, un-opsonized gram-negative bacteria also displayed an efficient clearance, which cannot be explained by the direct recognition of lipoteichoic acid (Broadley et al., 2016). Therefore, a “dual track clearance” mechanism consisting of parallel “fast” and “slow” clearance of circulating bacteria has been described (Broadley et al., 2016). Circulating bacteria (opsonized or not) are rapidly cleared by the liver KCs via CRIg and scavenger receptors, whereas the slower process of complement opsonization enables a second clearance step via platelet binding and phagocytosis by KCs, also using CRIg. Even though the liver captures and kills >90% of all circulating pathogens, a shift toward spleen clearance has been observed with growing particle size. This mechanism adds another layer to the efficient and fast clearance of circulating bacteria by the liver and spleen. Interestingly, CRIg has also been found to be a negative regulator of T-lymphocyte responses in tissues. CRIg can function as a coinhibitory molecule of the B7/CD28 superfamily, suppressing T-lymphocyte proliferation and cytokine production, thereby maintaining peripheral T-lymphocyte tolerance in healthy tissues (Vogt et al., 2006; Yuan et al., 2017; Munawara et al., 2019). Interestingly, this inhibitory function is regulated by CRIg internalization when bound to C3b or iC3b (e.g., opsonized target), allowing an adequate T-lymphocyte response to progress during tissue inflammation (Fearon et al., 1981; Sengeløv et al., 1994).

Murine KCs express CRIg and CR3 on the plasma membrane, while human KCs additionally express CR1 and CR4. However, none of these receptors on KCs are more involved in pathogen clearance than CRIg. The relationship between CRIg and CR3 was investigated in mice. Even though both receptors are expressed on KCs and share a common ligand, distinct modes of pathogen clearance have been observed. CRIg binds and internalizes opsonized pathogens independently of receptor crosslinking, additional activation stimuli or the presence of divalent cations, which are all indispensable requirements for CR3-mediated phagocytosis (Gorgani et al., 2008). Thus, CR3 contributes rather indirectly to pathogen clearance by the recruitment of neutrophils through their interaction with ICAM-1 (Gregory et al., 2002). The subcellular localization and intracellular trafficking of CRIg also differs from CR3: CRIg is mostly expressed on recycling endosomes where they aid in delivering membrane to the forming phagosome and ensure a sufficient supply of CRIg to the plasma membrane to mediate CRIg-dependent internalization (Helmy et al., 2006). In contrast, CR3 is located in secretory vesicles that fuse with the plasma membrane upon cytokine stimulation. CRIg is not degraded after particle internalization, instead, CRIg is recycled to the endosome pool prior or during phagosome-lysosome fusion. The signaling mechanism induced by CRIg activation is not known. CR3 and CRIg potentially share some intracellular mediators of the CR3 signaling pathway, as they are co-expressed on macrophages and share a common ligand, however, more studies are needed to clarify this.

The ability of CRIg to bind to the beta chain subunit of C3b abrogates the interaction of C3 and C5 convertases of the alternative pathway (Wiesmann et al., 2006). The potential immune regulatory role of CRIg has further been investigated and led to the development of a soluble CRIg-Fc fusion protein with enhanced complement inhibitory efficacy. Because CRIg only blocks complement activation of the alternative pathway, and not the classical or lectin pathway, the novel CRIg-Fc complement inhibitor was thought to have an effect on the progression of diseases in which the alternative pathway contributes greatly. Its benefits were confirmed in mouse models of arthritis, where CRIg-Fc injection caused a reduction of inflammation and bone loss compared to control mice, even when the disease was already established (Katschke et al., 2007). Also, lupus-prone MRL lymphoproliferation (MRL/lpr) mice showed significantly less skin lesions, proteinuria and kidney pathology when treated with CRIg-Fc (Lieberman et al., 2015), and it prevented local and remote tissue injury induced by ischemia-reperfusion (Chen et al., 2011). A novel CRIg/FH fusion protein, combining the extracellular domain of CRIg and the functional domain of factor H, was designed to inhibit both the classical and alternative complement pathway and displayed similar effects in ischemia-reperfusion injury and lupus nephritis (Qiao et al., 2018; Hu et al., 2019; Shi et al., 2019). All together, these results indicate the potential effective role of soluble CRIg proteins in clinical settings of intestinal and renal ischemia-reperfusion injury, SLE, inflammatory arthritis, autoimmune liver disease (Jung et al., 2012) and potentially other diseases in which the alternative pathway is involved.

### Complement Receptor 2 (CR2)

#### Receptor Characterization

CR2 (CD21) is a 145 kDa type 1 membrane bound glycoprotein that comprises 15–16 CCPs (depending on the alternative splicing of one exon), a transmembrane domain and a short 34 amino acid cytoplasmic tail (Figure 2) (Hannan et al., 2002). The receptor structure closely resembles CR1, but lacks a few N-terminal CCP repeats that are known to bind C3b/C4b. Instead, CR2 binds the ligands iC3b, C3dg, and C3d (Molina et al., 1994). In humans, CR2 binds the gp350/220 viral envelope protein of the Epstein-Barr virus (EBV) (Fingeroth et al., 1984), the immunoregulatory protein CD23 (Aubry et al., 1992) and interferon-alpha (Asokan et al., 2006). Szakonyi et al. provided substantial information on the structure of CR2 by determining the crystal structure of CCP1 and CCP2 in complex with C3d at 2.0 Å (Szakonyi et al., 2001). Human CR2 is expressed primarily on mature B-lymphocytes and FDCs, although a subset of peripheral and thymic T-lymphocytes and epithelial cells also express the receptor (Table 1).

#### CR2-Mediated Functions

CR2 exerts distinct functions depending on ligand binding: (1) On B-lymphocytes, CR2 promotes antigen receptor-mediated signal transduction by the formation of a B-lymphocyte co-receptor complex with the signaling protein CD19 and the tetraspanin CD81. Co-ligation of the B-cell receptor with CR2-CD19-CD81 complexes by C3d-coated antigens/immune complexes can significantly amplify signaling in B-lymphocytes and lower the threshold for B-lymphocyte activation by at least two orders of magnitude (Carter and Fearon, 1992). (2) On FDCs, CR2 mediates the retention of C3-coated antigens, presumably to enhance interactions with B-lymphocytes of the germinal center (Reynes et al., 1985). (3) As a receptor for CD23, one of the main functions of CR2 is the promotion of B-lymphocyte class switching and the increased production of IgE (Aubry et al., 1992). (4) EBV hijacks CR2 for B-cell infection; binding to CR2 initiates the entry of the virus in B-lymphocytes (Tanner et al., 1987).

CR2 is not directly implicated in adhesion or phagocytosis. However, there are indications that CR2 contributes to the pathogenesis of SLE. In patients with SLE, the expression of CR1 and CR2 on B-lymphocytes is decreased by 50% and a CR2 variant with three single nucleotide polymorphisms (SNPs) was associated with a 1.54 increased risk of SLE (Wilson et al., 1986; Wu et al., 2007). However, whether CR2 defects are the consequence or the drivers of the disease is not completely clear. In the mouse model of SLE (MRL/lpr), reduced expression of CR1 and CR2 occurred on B-lymphocytes before the clinical signs of SLE appear (Takahashi et al., 1997). Also, SNPs in the *Cr2* gene are sufficient for mice to develop an SLE-like disease (Boackle et al., 2001). Moreover, increased serum levels of antinuclear Abs and anti-DNA Abs have been found in Cr2^null^ mice (Wu et al., 2002). These results indicate a role for CR2 in SLE pathogenesis, however, one must keep in mind that these were obtained in mice, in which a single gene encodes for both CR1 and CR2. Human CR2 has been shown to bind DNA and chromatin in the absence of C3 opsonization, therefore, CR2 deficiency in SLE might also influence the development of autoimmunity in SLE through altered receptor interactions with DNA (Asokan et al., 2013).

### Complement Receptors CR3 and CR4

#### CR3 and CR4-Mediated Functions

Out of all complement receptors, CR3 (CD11b/CD18) is the most widely expressed and a highly versatile receptor. It is expressed by macrophages, monocytes, neutrophils, dendritic cells, NK cells and activated lymphocytes (Table 1) (Ho and Springer, 1982; Ross and Vetvicka, 1993). The importance of CR3 is highlighted by its contribution to both the recruitment of leukocytes (via adhesion) and phagocytosis of targets. CR4, which contains the CD11c α-chain instead, is highly expressed in monocytes, macrophages and DCs, where it mediates similar functions to CR3 (Torres-Gomez et al., 2020a). For a detailed description of CR3 and CR4 structure and motifs, see section β2-Integrin Family Complement Receptors CR3 and CR4.

CR3 and CR4 interact predominantly with iC3b to promote phagocytosis, a fragment that is generated from factor I-dependent cleavage of C3b (Beller et al., 1982; Keizer et al., 1987). In general, presence of iC3b-opsonized particles is not sufficient to induce CR3-mediated phagocytosis. CR3 requires inside-out activation which includes a receptor conformational change into the high-affinity “extended” and “open” state (E^+^H^+^) and receptor clustering in the membrane, together resulting in efficiently binding and internalizing iC3b-opsonized particles. Integrin clustering has been found to be indispensable for ligand binding and receptor signaling, with FcγR stimulation promoting CR3 aggregation in phagocytic cups by enhancing the receptor's lateral mobility (Jongstra-Bilen et al., 2003). Also, ligand binding to CR3 was enhanced in neutrophils with increased receptor clustering after PMA stimulation, with loss of clustering correlating with a loss in receptor activity (Detmers et al., 1987). The stimuli for inside-out signaling include inflammatory cytokines (TNF-α), chemokines, N-formylmethionine-leucyl-phenylalanine (fMLP), TLR agonists and adhesion to extracellular matrix (laminin, fibronectin) (Sampson et al., 1991). These lead to inside-out activation via the Rap1-RIAM-Talin pathway. Genetic ablation of these proteins leads to defects in complement-mediated phagocytosis, as illustrated by impaired adhesion, phagocytosis and ROS production by RIAM-deficient phagocytes *in vitro*, and signs of LAD in RIAM knockout mice *in vivo* (Klapproth et al., 2015; Torres-Gomez et al., 2020b). Inside-out signaling drives the phosphorylation of CD18 on serine residues, but not of the alpha chains (CD11b or CD11c), which are constitutively phosphorylated (Chatila et al., 1989; Fagerholm et al., 2006). These phosphorylations, although originating differently, are both required for CR3- and CR4-mediated leukocyte adhesion and phagocytosis.

Ligand binding initiates a phagocytic signal that promotes particle internalization via a signaling pathway that relies on well-defined molecular players, described in detail in section Introduction to Phagocytosis. It requires Arp2/3 and mDia-mediated actin polymerization, and Rho activity. Actin polymerization that drives the phagosomal cup expansion is coupled to integrins via the activity of tyrosine kinases (e.g., Src and Syk) and binding to talin and vinculin which create anchoring points for the force transmission from actin polymerization (Jaumouille et al., 2019). Besides iC3b, a wide range of unrelated ligands are also capable of interacting with CR3, including but not limited to fibrinogen, factor X, neutrophil inhibitory factor (NIF), collagen, denatured proteins and plastics (Yakubenko et al., 2002). Studies with inhibitory mAbs directed to multiple regions of this receptor showed that the I domain of CR3 contains multiple overlapping ligand binding sites which are responsible for the receptor's broad ligand specificity (Diamond et al., 1993). Within this domain, three amino acids (Phe^246^, Asp^254^, Pro^257^) were identified as critical for CR3-dependent ligand binding (Yakubenko et al., 2002). Although CR3 and CR4 interact with a number of unrelated ligands with no clear receptor consensus motif, the MIDAS of the I domain is apparently a common ligand recognition site for both receptors (Vorup-Jensen and Jensen, 2018). In addition to the I domain, a unique lectin domain, located C-terminally to the I domain, participates in the binding of microbial carbohydrates (e.g., beta-glucan) (Ross et al., 1987). The binding of cell wall carbohydrates to the lectin domain serves as an additional signal to mediate phagocytosis of iC3b-opsonized fungi (Cain et al., 1987). In addition, the lectin domain has been shown to be responsible for the non-opsonized phagocytosis properties of CR3, as demonstrated by the phagocytosis of non-opsonized zymosan (Le Cabec et al., 2002).

Although CR3 and CR4 share a high degree of homology, functional differences between these receptors are becoming progressively clearer. For instance, CR3 and CR4 bind iC3b differently (Xu et al., 2017). CR3 binds iC3b at two separate sites, which are distinct from the two iC3b-binding sites found in CR4. Moreover, CR3 has generally more affinity toward positively-charged ligands, such as major basic protein in the brain and the antimicrobial peptide LL-37. On the other hand, CR4 binds well to heparin and osteopontin, both highly negative molecules (Vorup-Jensen and Jensen, 2018). CR3 and CR4 may also differ in which function they perform preferentially in cells. Studies *in vitro* suggested CR3 as the main phagocytic receptor for iC3b-opsonized bacteria, whereas CR4 predominated as an adhesion molecule for monocytic cells (Erdei et al., 2019). In alveolar macrophages, CR3 mediated the majority of the attachment to C3-opsonized sheep erythrocytes, whereas CR4, although more abundant, had a minor role. That disparity was correlated to differences in plasma membrane motility of CR3 and CR4, with CR4 being less mobile (Ross et al., 1992). Also, there is only 56% homology between the cytoplasmic tail of CR3 α and CR4 α, indicating cytoplasmatic structural differences that might affect the binding of signaling molecules, possibly contributing to some distinct receptor functional properties (Ross et al., 1992).

Deficiencies in CR3 and CR4 recapitulate in many ways the deficiency of complement components, such as in the susceptibility to recurrent infections (Rosetti and Mayadas, 2016). These are of particular importance in CR3 and CR4 since the receptors not only control phagocytosis of opsonized material, but of unrelated ligands and also mediate the recruitment of leukocytes. CD11b deficiency leads to more severe sepsis and larger bacterial load in a model of murine cecal-ligation and puncture (Liu et al., 2014). Similarly, mice infected with *S. aureus* (Flick et al., 2004) or *S. pneumoniae* (Prince et al., 2001) have increased bacteremia and mortality when lacking a functional CR3. Deficiency in CD18 causes LAD-I, characterized by frequent life-threatening infections, elevated neutrophil numbers in the bloodstream, and impaired wound healing. Patients with LAD-I-related mutations in CD18 present reduced expression of CR3 and functional defects, such as low binding to iC3b, bovine serum albumin and fibrinogen (Hogg et al., 1999; Mathew et al., 2000). Some unexpected aspects of infection are also revealed in the absence of CR3 and CR4. Phagocytosis of the pathogen *C. neoformans* requires complement receptors, although it does not require complement opsonization (Taborda and Casadevall, 2002). Antibody mediated blockade of CR3 and CR4, or deficiency in CD18 impaired macrophage phagocytosis of the yeast significantly, which was triggered by glucuronoxylomannan molecules exposed at the yeast capsule. Moreover, the gram-negative bacteria *F. tularensis* subvert the complement system to foster bacterial survival. *F. tularensis* is phagocytosed by DCs in a C3-, CR3-, and CR4- dependent manner, however, this internalization mechanism stimulates proinflammatory cytokine production, intracellular bacterial growth and DC death instead (Ben Nasr et al., 2006).

Complement is also involved in the clearance of dead cells. C3 opsonization and CR3 are required for the clearance of apoptotic Jurkat cells by macrophages *in vitro*. Antibody blockade of C3 or CR3 was able to inhibit apoptotic cell clearance significantly, whereas CR4 blockade had a partial effect (Takizawa et al., 1996). An interesting observation by Mevorach et al. was that addition of serum to assays of apoptotic cell phagocytosis increased the uptake efficiency several fold (Mevorach et al., 1998). In this study, complement deposition was induced, among other factors, by exposed phosphatidylserine in apoptotic cells. Interestingly, it was shown that transfection of CHO cells with CR3 alone is sufficient to promote phagocytosis of apoptotic bodies by CHO cells. More recently, numerous studies implicated CR3 and CR4 also in the clearance of necrotic cells (Gaipl et al., 2001; Gullstrand et al., 2009). It was shown that complement factors C1q and C3 bound preferentially to necrotic cells over apoptotic cells *in vitro*, which drove their phagocytosis by macrophages. In addition, CR3 and CR4 present a variety of scavenging functions that are independent of opsonization, such as its binding to nucleic acids, glycosaminoglycans and denatured proteins (Vorup-Jensen and Jensen, 2018). Both receptors, but especially CR3, are hypothesized to assist in the prevention of autoimmunity and inflammation by promoting debris clearance. In line with this, genome-wide association studies have identified 3 SNPs that are strongly associated with the development of SLE (Nath et al., 2008; Faridi et al., 2017). These SNPs are located in the *ITGAM* gene and result in a variety of dysfunctions of CD11b, including reduced integrin activation, leukocyte adhesion, ligand binding and phagocytosis. Moreover, the defective CD11b variants cause an excessive production of type I interferon, which drives SLE development and severity (Faridi et al., 2017).

## Conclusion

In this review, the role of complement and complement receptors in the regulation of immunity and inflammation was discussed, with a focus on their function in leukocyte recruitment and phagocytosis in several tissues. Activation of complement anaphylatoxin receptors C3aR and C5aR mainly mediates chemotaxis of leukocytes to inflammatory sites for pathogen clearance or tissue regeneration. C5L2 is additionally involved in regulation of C5aR signaling, by exerting both immune suppressive as immune activating functions. Activation of anaphylatoxin- and other GPCRs during rolling is essential for further leukocyte transmigration into inflamed tissues, which is mediated by activation of CR3, CR4 and other integrins. After extension by inside-out and outside-in signaling, they participate in firm endothelial adhesion, diapedesis and leukocyte chemotaxis toward the inflammatory trigger. Within tissues, activated CR3 and CR4 are involved in phagocytosis by mainly interacting with iC3b-opsonized pathogens. Moreover, complement receptors CR1, CRIg and CR2 are interacting with several components of the complement cascade, as such contributing to complement-mediated phagocytosis and cell-type specific immune regulatory roles. The importance of a correctly functioning complement system is highlighted by diseases such as LAD and SLE, which are characterized by deficiencies in leukocyte extravasation and phagocytosis due to impaired complement molecules. Differences in complement proteins between mice and men and the differences in cells that express specific complement receptors in both species do not facilitate research on molecular pathways and partially explain remaining knowledge gaps. Examples are our lack of understanding on the mechanisms that allow CR3 to either stimulate phagocytosis or cell migration depending on the type of ligand bound. Novel therapies targeting the complement system have great beneficial potential in a number of kidney, brain and articular diseases, highlighting the significance of further research on complement receptor function and regulation.

## Author Contributions

SV, SC, and PM wrote the manuscript and prepared the figures. PP and PM provided critical input and corrected the manuscript. All authors contributed to the article and approved the submitted version.

## Conflict of Interest

The authors declare that the research was conducted in the absence of any commercial or financial relationships that could be construed as a potential conflict of interest.
